# Type 2 Respiratory Failure as an Initial Manifestation of Lambert–Eaton Myasthenic Syndrome Complicated by Paraneoplastic Autoimmune Encephalitis

**DOI:** 10.7759/cureus.78284

**Published:** 2025-01-31

**Authors:** Sota Uemura, Satoru Fujiwara, Tsuyoshi Sasada, Nobuo Kohara, Michi Kawamoto

**Affiliations:** 1 Department of Neurology, Kobe City Medical Center General Hospital, Kobe, JPN; 2 Department of Respiratory Medicine, Kobe City Medical Center General Hospital, Kobe, JPN

**Keywords:** acute respiratory failure, lambert-eaton myasthenic syndrome, nerve conduction study, paraneoplastic autoimmune encephalitis, paraneoplastic neurological syndromes

## Abstract

Lambert-Eaton myasthenic syndrome (LEMS) is a paraneoplastic or autoimmune neuromuscular disorder that typically presents with limb weakness or autonomic dysfunction. Here, we report a rare case of LEMS with acute type 2 respiratory failure as the initial symptom. A 79-year-old woman was admitted with acute disturbance of consciousness and type 2 respiratory failure. On admission, she presented with confusion, seizures, and respiratory acidosis requiring non-invasive positive pressure ventilation. Cerebrospinal fluid analysis revealed pleocytosis and elevated protein levels without infection. Electroencephalography showed nonspecific slowing, while imaging revealed no abnormalities. A nerve conduction study on day 10 confirmed LEMS, with findings of low-amplitude compound motor action potentials and facilitation on high-frequency stimulation. Coexisting autoimmune encephalitis was diagnosed based on clinical presentation and serum antibody positivity for voltage-gated calcium channels and gamma-aminobutyric acid B receptors. Positron emission tomography/CT identified small-cell lung cancer, confirmed by biopsy. The patient and family declined invasive cancer therapies. Then, the patient passed away on day 60.

This case highlights the diagnostic challenge of atypical presentations of paraneoplastic neurological syndromes, including LEMS and autoimmune encephalitis. Early recognition of LEMS in patients with unexplained type 2 respiratory failure through nerve conduction studies is critical. This report underscores the importance of considering paraneoplastic etiologies in patients with multiple neurological syndromes.

## Introduction

Lambert-Eaton myasthenic syndrome (LEMS) is a paraneoplastic or primary autoimmune disorder of the neuromuscular junction, typically characterized by gradually progressive proximal limb weakness or autonomic dysfunction as the initial symptom. Early diagnosis is crucial because identifying and treating the underlying malignancies is essential for optimal management, but it can be challenging, particularly when the clinical presentation is atypical [1].

Small-cell lung cancer (SCLC), the most common tumor associated with LEMS, is also known to be associated with other paraneoplastic neurological syndromes (PNSs). Among them, autoimmune encephalitis is characterized by acute to subacute progressive symptoms caused by central nervous system dysfunction associated with antibodies targeting neuronal cell surface and synaptic proteins [2].

Here, we report a rare case of the coexistence of LEMS and autoimmune encephalitis presenting with acute and severe type 2 respiratory failure as the initial symptom. In this patient, both voltage-gated calcium channel (VGCC) and gamma-aminobutyric acid B (GABA-B) receptor antibodies associated with SCLC were positive. This report highlights the significance of considering LEMS in the differential diagnosis of acute type 2 respiratory failure to avoid missing appropriate treatment opportunities.

## Case presentation

**Figure 1:**
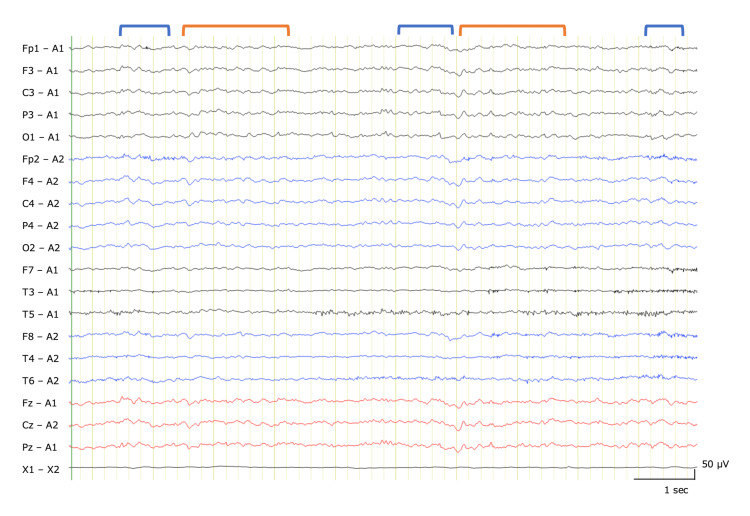
Electroencephalography findings of the patient on day 4 The background activity was poorly organized theta waves (6–7 Hz) (marked in orange), and there were intermittent diffused theta–delta waves (2–4 Hz) (marked in blue).

A 79-year-old woman was transferred to the hospital because of an acute disturbance of consciousness. Before symptom onset, the patient was generally well without apparent respiratory distress, although her family noticed mildly confused speech over the preceding days. On admission, the patient was confused and disoriented, had a Glasgow coma scale score of 10/15, and experienced a brief convulsive seizure. The patient’s respiratory rate was elevated (30/min), and oxygen saturation was 89% on room air. An arterial blood gas analysis indicated respiratory acidosis (pH 7.27, PaCO₂ 54.2 mmHg), requiring non-invasive positive pressure ventilation (NIPPV). There were no signs of wheezing, peripheral edema, finger clubbing, lymphadenopathy, or other notable findings. There was no muscle atrophy in the trunk, extremities, or tongue. The blood test revealed a sodium level of 138 mEq/L, with no other significant abnormalities observed. A cerebrospinal fluid (CSF) analysis revealed pleocytosis (21 cells, 95% lymphocytes) and elevated protein levels (60 mg/dL). However, the glucose level did not decrease. The FilmArray Meningitis/Encephalitis Panel® and the CSF Gram stain yielded negative results. Electroencephalography revealed a slow background and intermittent diffuse delta waves without rhythmic or periodic patterns (Figure 1). Brain MRI and chest CT showed no apparent abnormalities, such as findings of emphysematous lungs indicative of chronic obstructive pulmonary disease severe enough to cause type 2 respiratory failure.

**Figure 2:**
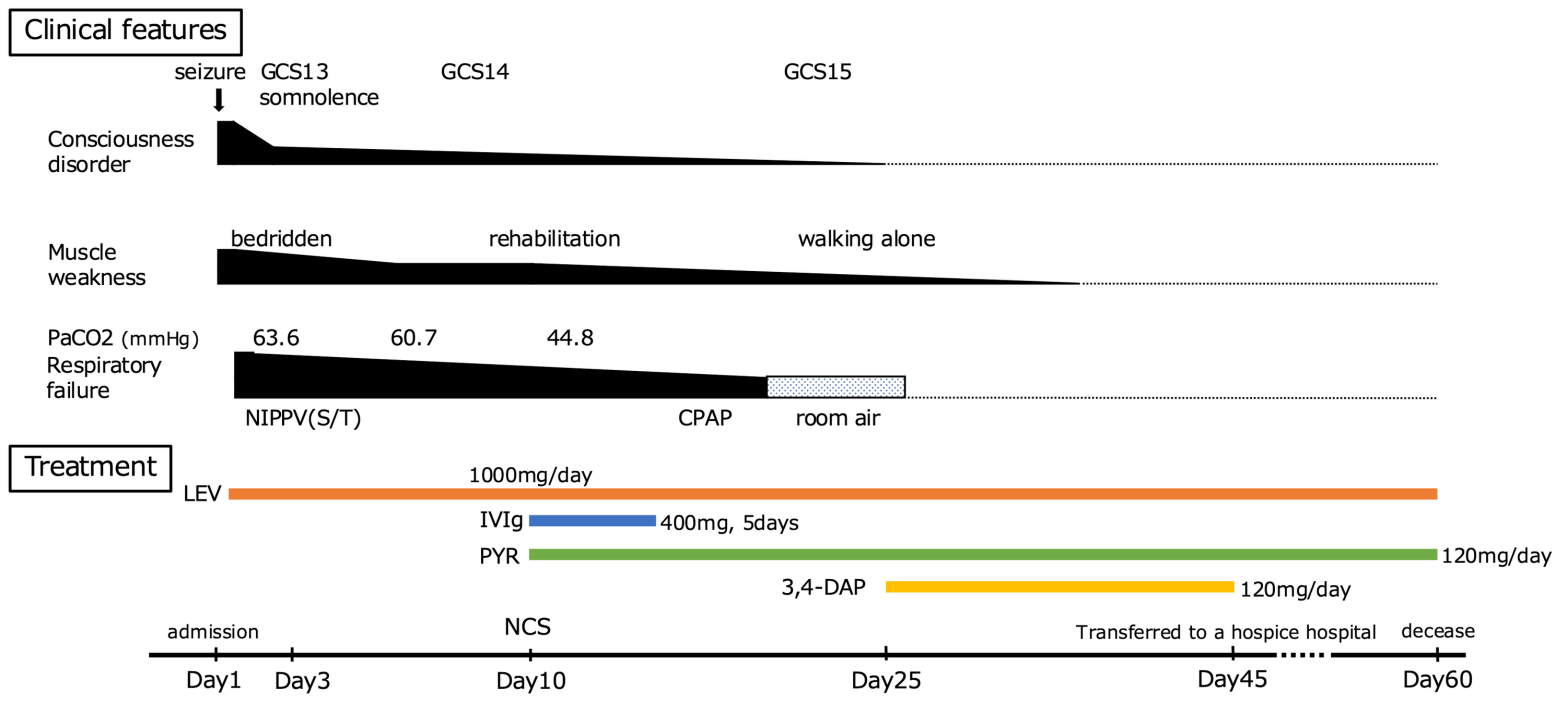
Clinical course and treatment GCS: Glasgow coma scale, NIPPV: non-invasive intermittent positive pressure ventilation, CPAP: continuous positive airway pressure, LEV: levetiracetam, IVIg: intravenous immunoglobulin, PaCO2: partial pressure of carbon dioxide, PYR: pyridostigmine, 3,4-DAP: 3,4-diaminopyridine, NCS: nerve conduction study

**Figure 3:**
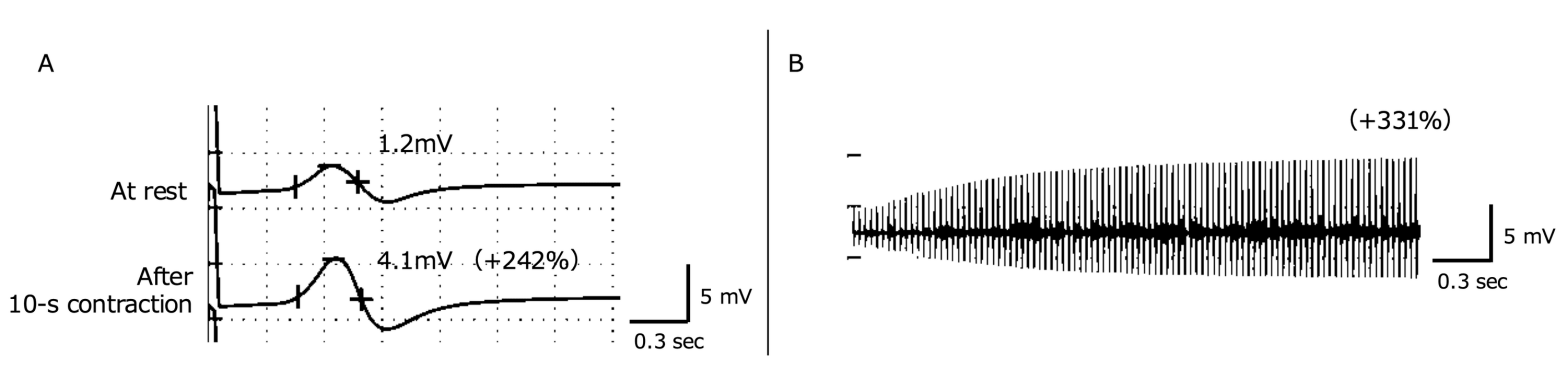
Ulnar motor NCS results recorded from the abductor digiti minimi with stimulation at the wrist (A) Compound muscle action potential amplitude at rest and after 10 seconds of maximum construction. (B) 20 Hz response for five seconds. (+%) represents an incremental response with 10 seconds of exercise or 20 Hz stimulation. NCS: nerve conduction study

**Figure 4:**
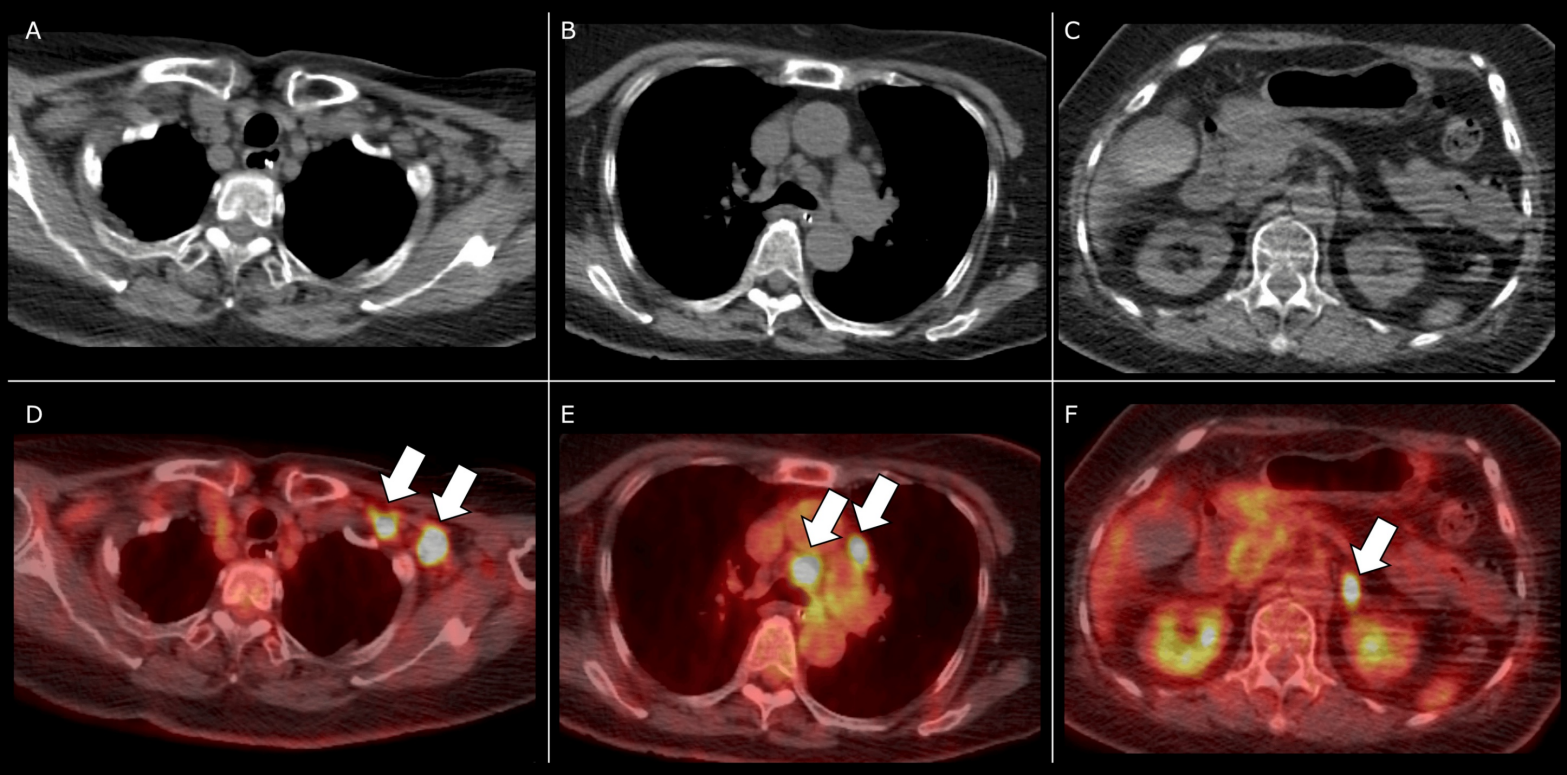
CT and positron emission tomography/CT There was intensive 18F-fluorodeoxyglucose uptake at mediastinal lymph nodes, supraclavicular lymph nodes, and the left adrenal gland (arrow). CT: computed tomography

Levetiracetam was administered for a first-time seizure, suspected to be an acute symptomatic seizure secondary to acute encephalitis, given the increased CSF cell count. The level of consciousness improved rapidly; however, respiratory failure requiring NIPPV persisted (Figure 2) and slight limb muscle weakness, suggesting the possibility of an overlapping condition in addition to encephalitis. A nerve conduction study (NCS) was performed on day 10 as part of a detailed evaluation. It revealed low-amplitude compound motor action potentials with the facilitation of brief exercise and high-frequency stimulation, consistent with LEMS (Figure 3). The serum P/Q-type VGCC antibody was found to be positive later. We diagnosed the patient with coexisting autoimmune encephalitis and LEMS and initiated intravenous immunoglobulin and oral pyridostigmine on day 10. Both symptoms improved significantly within two weeks of starting treatment. By day 25, the patient was weaned from NIPPV. The patient remained stable without symptom recurrence following oral 3,4-diaminopyridine therapy, which was administered with the ethics committee's approval, as it was not covered by insurance in our country. Positron emission tomography/CT revealed intensive 18F-fluorodeoxyglucose uptake at mediastinal lymph nodes that were not visible on the initial CT (Figure 4), and a biopsy showed pathological findings consistent with SCLC. The result of an encephalitis panel test (encephalopathy, autoimmune/paraneoplastic evaluation, serum by Mayo Clinic Laboratories, Rochester, MN, USA) showed that the serum GABA-B receptors are also positive on day 27, supporting a diagnosis of LEMS with paraneoplastic autoimmune encephalitis. Invasive treatment for SCLC was not pursued per the wishes of the patient and family. The patient was transferred to another hospital and died on day 60 (Figure 2).

## Discussion

This case highlights the unusual presentation of LEMS with paraneoplastic autoimmune encephalitis, manifesting as type 2 respiratory failure. LEMS often presents with a gradual onset of limb weakness, autonomic symptoms, and respiratory failure, as the initial symptom is exceptionally rare. To our knowledge, only one prior case of LEMS presenting with acute respiratory failure has been reported [3]. Respiratory symptoms, as comorbid manifestations, typically appear during the advanced stages in severely affected patients. In this case, subclinical respiratory impairment due to LEMS may have become clinically apparent when compounded by encephalitis-related seizures and impaired consciousness, leading to the unusual clinical course as LEMS observed in this case. Additionally, the coexistence of two PNSs, LEMS and autoimmune encephalitis, within a short timeframe is uncommon. Although various onconeural antibodies are known to be associated with SCLC [4], concurrent manifestations of multiple PNSs are rarely documented [5-7].

Type 2 respiratory failure can have various causes; for instance, neuromuscular diseases are listed in Table 1. We propose that respiratory fatigue caused by LEMS, combined with central hypoventilation associated with GABA-B receptor encephalitis, likely interacted to result in type 2 respiratory failure in this patient ultimately. The frequency of such occurrences is unclear, but in unexplained type 2 respiratory failure cases, NCS should be actively performed to evaluate neuromuscular disorders, including LEMS.

**Table 1:** List of various extrapulmonary conditions that causes type 2 respiratory failure This is a list of extrapulmonary conditions, focusing on items related to neurological diseases. The diseases diagnosed in this case are indicated in bold.

Central nervous system disorders
Brainstem lesions (e.g., stroke, tumors, trauma)
Central nervous system depressants (e.g., opioid, benzodiazepine)
Traumatic brain injury
Encephalopathy
Metabolic encephalopathy (e.g., hepatic encephalopathy, uremic encephalopathy)
Hypoxic-ischemic encephalopathy
Toxic encephalopathy (e.g., carbon monoxide poisoning)
Encephalitis
Viral encephalitis (e.g., herpes simplex virus, arboviruses)
Autoimmune encephalitis
Spinal cord disorders
Cervical spinal cord injury
Acute transverse myelitis
Neuromuscular junction disorders
Myasthenia gravis (including myasthenic crisis)
Lambert-Eaton myasthenic syndrome
Peripheral nervous system disorders
Guillain-Barré syndrome
Critical illness polyneuropathy
Diaphragmatic paralysis due to peripheral nerve involvement (e.g., phrenic nerve injury)
Neuromuscular disorders
Muscular dystrophy
Congenital myopathies
Acute myopathy
Amyotrophic lateral sclerosis

## Conclusions

This case emphasizes the importance of considering LEMS as a potential cause of type 2 respiratory failure, particularly in the context of PNSs. Early recognition and prompt diagnostic evaluations, such as NCS, are crucial for guiding treatment and uncovering underlying malignancies. Clinicians should remain vigilant for atypical presentations of PNSs, especially when multiple syndromes coexist.
